# When Do Low Status Individuals Accept Less? The Interaction between Self- and Other-Status during Resource Distribution

**DOI:** 10.3389/fpsyg.2016.01667

**Published:** 2016-10-25

**Authors:** Philip R. Blue, Jie Hu, Xueying Wang, Eric van Dijk, Xiaolin Zhou

**Affiliations:** ^1^Center for Brain and Cognitive Sciences, Peking UniversityBeijing, China; ^2^School of Psychological and Cognitive Sciences, Peking UniversityBeijing, China; ^3^Department of Social and Organizational Psychology, Leiden UniversityLeiden, Netherlands; ^4^Leiden Institute for Brain and Cognition, Leiden UniversityLeiden, Netherlands; ^5^Key Laboratory of Machine Perception (Ministry of Education), Peking UniversityBeijing, China; ^6^Beijing Key Laboratory of Behavior and Mental Health, Peking UniversityBeijing, China; ^7^PKU-IDG/McGovern Institute for Brain Research, Peking UniversityBeijing, China

**Keywords:** social status, ultimatum game, social hierarchy, fairness, acceptance

## Abstract

In real-world social interactions, social status influences responses to resource distribution. However, the way in which one’s own social status interacts with another’s status to influence responses to resource distribution is far from clear. In the current study, we dynamically manipulated participants’ social status and then asked participants to act as recipients in the ultimatum game (UG) along with proposers whose social status was made known to the participants. Experiment 1 used a between-participants design in which the participants were assigned as being of either high or low status according to their performance in a math competition (i.e., rank-inducing task). In Experiment 2, social status was manipulated within-subjects using the same rank-inducing task, with rounds of UG interleaved between rank-inducing sessions. Findings from the two experiments showed that both self-status and other-status influenced responses to UG offers, as participants were more likely to accept low offers from high status than low status proposers; this effect was particularly robust for low status participants when compared with high status participants. These findings suggest that, in comparison with individuals in high status, individuals in low status are more willing to accept low offers during resource distribution and are more affected by other-status considerations.

## Introduction

Social hierarchies exists in almost all social species, ranging from ants and fish (Bshary et al., 2014) to humans. In more basic social hierarchies, each member’s place in the hierarchy is based along a power dimension that often involves the use of dominance (Magee and Galinsky, 2008); in more complex social hierarchies, such as those found in humans, each member’s place in the hierarchy is determined in a multi-faceted fashion, as it is not only based on power, but also on social status, which refers to prestige, competence, or respect in a relevant dimension or field (Henrich and Gil-White, 2001). Much of the work on status-related hierarchy centers on social class (also referred to as socioeconomic status, Adler et al., 2000; Kraus et al., 2009), which refers to one’s economic, professional, or educational standing. However, given the common mixture of power and social class, it is difficult to clearly delineate the effect of status from power, which results in an impoverished understanding of the effects of social status by itself.

Given the complexity of social hierarchies and the abundance of potential confounding factors such as feelings of power when using social class measures such as annual salary or education level, researchers analyzing the effect of social status often turn to controlled laboratory settings to prime social status (Zink et al., 2008). In these types of procedures, researchers manipulate the social status of participants by having them complete a rank-inducing task (e.g., trivia quiz, Ball et al., 2001; Albrecht et al., 2013), after which they give the participants a relative rank on the measured dimension in comparison with other participants, which is often indicated using stars (Ball et al., 2001; Zink et al., 2008; Hu et al., 2014, 2016). The use of stars is effective in indicating social status given their pervasive use in online shopping websites, videogames, and the military. This type of status, in which the participant is judged as “more” or “less” competent than other players, is a well-accepted tool for priming feelings of social status (Zink et al., 2008). Importantly, previous work shows that the effect of rank-induced status on responses to resource distribution is similar to the effect of status differences between men and women (Eckel and Grossman, 2001) and between high and low status African peasants (D’Exelle et al., 2009) on responses to resource distribution.

Past research on social status demonstrates the importance of social status during resource distribution, with low status individuals demanding less in bargaining situations than high status individuals (Ball et al., 2001; Albrecht et al., 2013; Hu et al., 2014, 2016). However, these studies have shortcomings that prevent a comprehensive understanding regarding the interaction between one’s own status and others’ status and its effect on economic decision-making. Albrecht et al. (2013) measured satisfaction ratings of disadvantageous, equitable, and advantageous payoffs between the participant and another hypothetical participant of inferior, similar, or superior status and found that individuals in inferior status perceived disadvantageous inequality payoffs as more satisfactory than superior status individuals. However, it is unclear to what extent the feelings of satisfaction can directly map onto actual economic decisions. Moreover, this study focused on the relationship between middle status participants and superior/inferior ranked partners, which does not allow for an investigation into the potential behavioral differences in participants of the lowest or highest status. Ball et al. (2001) did measure the effect of having high or low status in bargaining situations and found that low status participants demanded less than those in high status. However, this study separated low and high status by role (i.e., buyer and seller), which limits the amount of information regarding the potential interaction between self and other status across roles. Similarly, our previous research measuring the effects of social status on acceptance of low and high offers in the ultimatum game (UG) found that participants in low status were more likely to accept low offers than participants in high status (Hu et al., 2014, 2016). However, given that participants did not know their partner’s status, it is unclear how self and other status may interact to affect responses to low and high offers.

One overarching question in the above-mentioned studies is that they did not manipulate the participants’ and the party’s status simultaneously, making it unclear whether individuals in low status were more willing to accept less of the pie in general or if their acceptance took into account the social status of other parties involved in the resource distribution. The lack of a systematic understanding of the interaction between self- and other-status on feelings toward resource distribution is critical not only because one’s own and others’ social status rarely exist independently in the real world, but also because people can accurately encode one’s own and others’ social status within minutes of meeting each other (Anderson and Kilduff, 2009), and adjust their behavior accordingly.

One of the most widely used research tools for measuring individuals’ responses to resource distributions is UG (Güth et al., 1982). In UG, a proposer is given a set amount of money and asked to divide it with another player, the recipient. If the recipient accepts the offer, then the two receive the allocated amount; if the recipient rejects the offer, the two players receive nothing. Traditional economic theory suggests that proposers should offer the lowest acceptable amount, while the recipient should accept any non-zero offer. However, this type of economic mindset is rarely found in actual experimental settings, as proposers tend to divide the money evenly, and the recipients’ acceptance rate of offers increases as a function of the offer level. Behavior in UG reflects not only fairness preferences but also strategic decision-making between two parties (Rabin, 1993). Importantly, previous studies have shown that the relationship between the two parties affects behavior in UG (Eckel and Grossman, 2001; Yu et al., 2015) or similar games (Wu et al., 2011).

Due to the lack of research on the interaction between one’s own and others’ social status during resource distribution, we turn to social class research to inform our hypotheses regarding the effects of self- and other-status on responses to resource distribution. On the one hand, a wide array of findings demonstrate that one’s own social status affects social interaction. In comparison with individuals with high social class, individuals with low social class are more perceptive and sensitive to the feelings and expressions of others (Kraus et al., 2010) and are more attuned to socially relevant and/or potentially threatening stimuli (Muscatell et al., 2012). Moreover, when compared with individuals in high social class, individuals in low social class are more compassionate and empathic to the needs of others (Kraus et al., 2012) and have been found to engage in more prosocial behavior such as generosity, charity, trustworthiness, and helping behavior, and in less selfish or destructive behavior such as breaking laws and social norms (Piff et al., 2010, 2012). On the other hand, a second line of research suggests the importance of other-status processing. For example, in situations that require unspoken coordination between two individuals, individuals of different social status coordinate more effectively than individuals of similar social status (De Kwaadsteniet and van Dijk, 2010). In addition, rhesus monkeys will give up sugary liquid reward to view high status monkeys (Deaner and Khera, 2005), and humans remember better and focus more attention on high status faces than low status faces (Ratcliff et al., 2011). The natural reward for accurately distinguishing high status others arises from the potential not only to obtain their protection but also to learn their ways (Tomasello et al., 1993, 2012).

The division in the potential effects of one’s own social status and others’ social status on decision-making allows for two potential hypotheses to emerge: (1) Participants’ acceptance of low UG offers increases as a function of proposer’s social status, regardless of one’s own social status (i.e., Proposer’s Status Hypothesis). In the current study, support for this hypothesis would be manifested by participants showing increased acceptance rates of low UG offers for high status proposers than for low status proposers, regardless of the participant’s own status. (2) Participants’ acceptance of low UG offers is most affected by other’s status when own status is low, which is supported by research showing that in situations that require coordination between two individuals, low status individuals tend to defer to high status others but do not do so for same-status others and by research on social class showing that individuals occupying a low social class are more sensitive to social information about other people (i.e., Interactive Status Hypothesis; De Kwaadsteniet and van Dijk, 2010; Kraus et al., 2010, 2012). Support for this hypothesis would be manifested by participants in low status showing a greater difference in acceptance rates of low UG offers by low and high status proposers than participants in high status.

**FIGURE 1:**
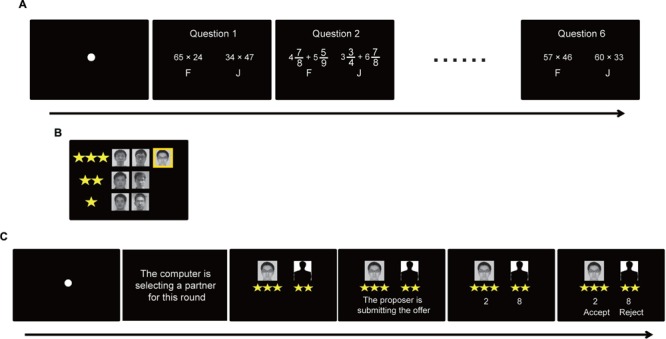
**Schematic diagram of the experiment.** Each experiment consisted of two sessions: the rank-inducing session and the UG session. In the rank-inducing session, the participants completed several time-constrained math questions together with seven other players by selecting which of the 2 arithmetic expressions was greater in value as quickly as possible **(A)**. After the rank-inducing session, participants then viewed status information based on his/her performance relative to others **(B)**. The participant’s photo was highlighted with a yellow background. In the UG session **(C)**, the participant was shown his/her own photo and social status information along with an anonymous silhouette and the social status information of the proposer before each UG offer was presented.

To address these hypotheses, in the current study we asked the participant to compete against seven other participants (confederates) in an interactive rank-inducing task (i.e., math competition) to dynamically manipulate both the participant’s and the opponents’ social status. All participants were raised in China, which places a very strong emphasis on the development of math skills from a young age, implying that a math-related ranking is especially relevant to one’s view of the self. In the math competition task (**Figure 1A**), the participants were given a maximum of 10 s for each question to indicate which math expression had a greater value, and the one who exhibited higher accuracy and lower response time attained a higher rank (**Figure 1B**). Afterward, participants acted as recipients in UG with proposers of varying social status (**Figure 1C**).

In addition, given that social status is a relative construct, it is extremely common for one’s social status to change from one situation to the next. In order to control for the potential effects of changes in social status on responses to resource distribution, participants’ self-status was a between-participant factor in Experiment 1 and a within-participant factor in Experiment 2.

## Experiment 1

### Method

#### Participants

To determine the sample size, we used G*Power 3 software (Faul et al., 2007), which showed that we needed a sample size of at least 92 for this study to have adequate power (1 – β > 0.80) to detect a medium-size effect (*f* = 0.20). One hundred and two undergraduate and graduate students (59 females) from local universities in Beijing participated in the study. Each participant was informed that the basic payment was around 30 Chinese yuan (about 5 USD) and additional monetary reward would fluctuate with their performance in the experiment (based on the random selection of 10 UG trial results). On average, participants received around 50 Chinese yuan (about 8 USD). Informed consent was obtained from each participant before the test. The experiment was conducted in accordance with the Declaration of Helsinki and was approved by the Ethics Committee of the School of Psychological and Cognitive Sciences, Peking University.

#### Design and Procedure

Experiment 1 had a 2 × 2 × 2 mixed design, with the first factor referring to the participants’ own social status (self-status: low vs. high) as a between-participant factor, the second factor referring to the proposer’s social status (other-status: low vs. high) as a within-participant factor, and the third factor referring to UG offer level (low vs. high) as a within-participant factor. The information of social status was given as a set of stars, where three stars represented high status, two stars represented middle status, and one star represented low status. UG offers equal to or less than 3 out of 10 yuan were operationally defined as “low,” while any offer greater than 3 yuan was defined as “high,” and no indication of “high” or “low” offers was expressed to the participant. Past research using a similar paradigm has shown that participants’ self-reported minimum acceptable UG offers range from 3.0 (*SE* = 0.06) to 3.4 (*SE* = 0.06) yuan (Hu et al., 2016). As a result, we used 3 yuan as a cutoff for “low” and “high” UG offers.

Upon arriving at the laboratory, the participant shortly met a same-sex confederate and was told that the two of them were going to play as recipients in the UG along with six same-sex strangers who would play as proposers and who had ostensibly arrived earlier and were getting prepared in another room. The participant never actually met the six proposers face-to-face and only saw pictures of their faces during the experiment. The purpose of such a setting was twofold: to increase the interactivity and credibility of the whole experiment, and to avoid a reputation-building effect in UG.

The experiment consisted of two tasks. The first was a rank-inducing task, which was referred to as the “rank-inducing session” (Zink et al., 2008; Boksem et al., 2012). This session had 12 time-constrained math questions (10 s/question), half of which were easy to solve, and half of which were difficult to solve (**Figure 1A**). The reason for using both easy and difficult arithmetic expressions was to be able to believably manipulate participant ranking by assuring the participant’s achievement of correct and incorrect responses. The participant’s task was to compare two arithmetic expressions and select which was greater in value by pressing the corresponding response key. There were two types of expressions, complex fraction addition (e.g., 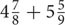) and two-digit multiplication (e.g., 34 × 46). Ranking was based on question accuracy and response time, with an emphasis on accuracy to avoid participants guessing quickly to increase time scores. The participant received his or her rank after completing the rank-inducing task (**Figure 1B**). To avoid any confounding effect of social comparison between the participant and the other recipient (confederate), we only presented the ranking information of the participant and that of the other six participants who would act as proposers in the subsequent UG.

The second task was UG (**Figure 1C**). Each trial began with the presentation of a fixation sign against a black background. Then the sentence “The computer is selecting a partner for this round” in Chinese was presented, suggesting to the participant that one of the six proposers was randomly selected by the computer in the current trial. Then the participant’s own portrait and a faceless silhouette were presented on the left and right side of the screen respectively. The positions of these two figures were counterbalanced over trials. At the same time, the rank information of the participant and that of the proposer which was paired with the participant in the current trial (denoted by a set of stars) were presented beneath the corresponding facial portrait/silhouette. Subsequently, the sentence “The proposer is submitting the offer” in Chinese was presented on the lower part of the screen indicating to the participant that the paired proposer was distributing the 10 yuan between them. Then, the proposer’s division scheme, with the amount for each player beneath the corresponding portrait/silhouette, was revealed. After receiving the offer, two options, “accept” and “reject,” appeared on the left and right side of the screen respectively, with their positions randomly exchanged over trials. The participant was asked to make the “accept” or “reject” decision by pressing the corresponding key on the keyboard. The participant was reminded that the proposers made their decisions individually and independently and that his/her decisions would not be revealed to them during the experiment.

The experiment was administered using Presentation software (Neurobehavioral System, Inc.) to control the arrangement and timing of stimuli. For each level of status, the amount for the participant in the low offer condition was drawn randomly from a Gaussian distribution with a mean of 2 and standard deviation of 0.5; the amount for the participant in the high offer condition was drawn randomly from a Gaussian distribution with a mean of 4 and standard deviation of 0.5. Low UG offers to the participant were less than or equal to 3 out of 10 yuan and ranged from 0.5/9.5 to 3/7; high UG offers to the participant were greater than 3 out of 10 yuan and ranged from 3.5/6.5 to 5.5/4.5. The number before the slash denoted the amount offered to the participant and the number after the slash denoted the amount allotted to the proposer. Unknown to the participant, all UG offers were predetermined by a computer program and pseudo-randomized with the restriction that no more than 3 consecutive trials were of the same offer level. There were four critical (high other-status/low UG offer, high other-status/high UG offer, low other-status/low UG offer, and low other-status/high UG offer) and two filler (middle other-status/low UG offer, and middle other-status/high UG offer) conditions in total, and 12 trials for every condition.

Before the formal test, participants performed 10 trials of the math competition task and 10 trials of UG to become familiar with the two tasks. To check the manipulation of social status, after the experiment, the participant was asked to indicate on a seven-point Likert Scale to what extent he/she perceived his/her status as higher (superior)/lower (inferior; 1 = much lower/more inferior, 7 = much higher/more superior) than the other players in the game. In order to confirm the usage of 3 yuan as a cutoff for the operational definition of “low” and “high” UG offers, after the experiment, participants indicated their minimal acceptable UG amount (out of 10 yuan). Finally, to measure participants’ fairness expectations, participants were asked to indicate what amount of UG offer (out of 10 yuan) would be considered a fair amount for each proposer status level.

Given the importance of emotions on decisions to reject in UG (Xiao and Houser, 2005; Harlé and Sanfey, 2007), after the experiment participants were asked to report on a five-point Likert scale (1 = not at all, 5 = very strongly) the extent to which they felt negative and positive emotions both when receiving their ranking information in the math competition task and during UG. The negative emotions included the following dimensions: irritable, uneasy, nervous, uncomfortable, angry, and shameful; the positive emotions included the following dimensions: interested, energetic, proud, inspired, determined, excited, happy, satisfied, and superior. At the end of the experiment, the participant was paid, debriefed, and thanked.

### Results

Among the 102 participants, eight claimed that they disbelieved the setup of the experiment. These participants were excluded from data analysis, leaving 94 participants (low self-status group: *n* = 47, 28 females, mean age 21.1 years, *SD* = 2.2; high self-status group: *n* = 47, 26 females, mean age 21.0 years, *SD* = 2.4) for the following analysis.

#### Manipulation Checks

The post-experiment questionnaire suggested that the number of stars used to denote the participants’ rank in the math competition task strongly influenced their perception of social status. A one-way (star ranking: 3 vs. 1) ANOVA on the perceived own status showed a significant main effect of star ranking, *F*(1,92) = 7.31, *p* = 0.008. The participants perceived themselves to be in higher status when they obtained three stars (high status) in the math competition task (mean ± SE, 4.45 ± 0.23, CI = [4.00, 4.90]) than those who obtained one star (3.51 ± 0.27, CI = [2.98, 4.04]).

The manipulation of other-status affected the participants’ self-reported minimal acceptable amount in UG (out of 10 yuan). The minimum acceptable offer was significantly higher when the offers were from low-status proposers (3.44 ± 0.12, CI = [3.19, 3.68]) than from high-status proposers (3.05 ± 0.11, CI = [2.84, 3.26]), *F*(1,92) = 12.80, *p* < 0.001, $\eta_{p}^{2}$ = 0.12. There was neither a significant main effect of self-status, *p* = 0.445, nor an interaction between self-status and other-status, *p* = 0.288. Self-reported minimal acceptable UG offer amount ranged from 3.05 to 3.44 out of 10 yuan, which is in line with previous research and in support of using 3 yuan as a cutoff for “low” and “high” UG offers.

The fairness expectations of participants in the high self-status group (5.18 ± 0.09, CI = [5.00, 5.36]) were higher than those in the low self-status group (4.52 ± 0.09), CI = [4.34, 4.69], *F*(1,92) = 28.59, *p* < 0.001, $\eta_{p}^{2}$ = 0.24. And participants’ fairness expectations were higher for low-status proposers (5.13 ± 0.09, CI = [4.95, 5.31]) than for high-status proposers (4.57 ± 0.08, CI = [4.41, 4.74]), *F*(1,92) = 21.80, *p* < 0.001, $\eta_{p}^{2}$ = 0.19. There was no significant interaction between self-status and other-status on fairness expectations, *p* = 0.292. These results suggest that the perceived fairness during resource distribution was modulated by both self-status and other-status.

Finally, social status also affected participants’ experience of emotions. We averaged scores on different dimensions to give overall scores for the negative and positive emotions. When receiving the ranking information, participants in the high self-status group (3.24 ± 0.11, CI = [3.03, 3.45]) experienced more positive emotions than the low self-status group (2.11 ± 0.10, CI = [1.90, 2.32]), *F*(1,92) = 57.51, *p* < 0.001, and participants in the low self-status group (1.90 ± 0.10, CI = [1.71, 2.10]) experienced more negative emotions than the high self-status group (1.60 ± 0.08, CI = [1.43, 1.76]), *F*(1,92) = 5.67, *p* = 0.019. These findings suggest that the math competition and social status ranking were meaningful to the participants, as participants who attained high status experienced more positive emotions than participants who attained low status.

Additionally, during UG, there was a very marginal difference in experienced positive emotions, *p* = 0.086, and no difference in negative emotions, *p* = 0.696, between the high self-status group and the low self-status group. This finding suggests that any effects of social status on behavior in UG should be attributed primarily to social status and not to the emotions associated with the experience of high or low status *per se*.

#### Behavioral Results

**FIGURE 2:**
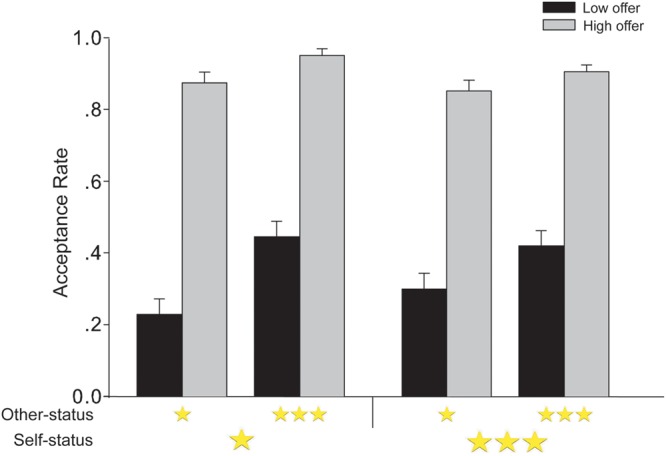
**The acceptance rate in Experiment 1 depicted as a function of self-status, other-status, and UG offer level.** One star = low status; three stars = high status. Error bars represent standard errors of the means.

We performed a 2 (other-status: high vs. low) × 2 (offer level: low vs. high) repeated measures ANOVA with self-status (high vs. low) as a between-participant factor on participants’ acceptance rates for different offers in UG (**Figure 2**). This analysis revealed a significant main effect of offer level, *F*(1,92) = 481.85, *p* < 0.001, $\eta_{p}^{2}$ = 0.84, with the acceptance rate for low offers (0.35 ± 0.03, CI = [0.29, 0.41]) being lower than for high offers (0.90 ± 0.02, CI = [0.87, 0.93]). The main effect of other-status was also significant, *F*(1,92) = 62.43, *p* < 0.001, $\eta_{p}^{2}$ = 0.40, indicating that the acceptance rate was higher for high status proposers (0.68 ± 0.02, CI = [0.64, 0.72]) than for low status proposers (0.57 ± 0.02, CI = [0.52, 0.61]). There was also a significant interaction between other-status and offer level, *F*(1,92) = 23.61, *p* < 0.001, $\eta_{p}^{2}$ = 0.20. Simple effects tests showed that acceptance rates for low offers were higher when the offers were from high status proposers (0.43 ± 0.03, CI = [0.37, 0.49]) than from low status proposers (0.27 ± 0.03, CI = [0.20, 0.33], *p* < 0.001, $\eta_{p}^{2}$ = 0.44), and this effect was smaller for high offers (high other-status: 0.93 ± 0.01, CI = [0.90, 0.96], low other-status: 0.86 ± 0.02, CI = [0.82, 0.91], *p* < 0.001, $\eta_{p}^{2}$ = 0.15). The main effect of self-status on acceptance rate was not significant, *p* = 0.885.

The analysis revealed a significant interaction between self-status and other-status, *F*(1,92) = 4.01, *p* = 0.048, $\eta_{p}^{2}$ = 0.04. Further tests revealed that participants in the low self-status group more frequently accepted offers from high status proposers (0.70 ± 0.03, CI = [0.65, 0.75]) than from low status proposers (0.55 ± 0.03, CI = [0.49, 0.62], *p* < 0.001, $\eta_{p}^{2}$ = 0.35), and this effect was smaller for participants in the high self-status group (high other-status: 0.66 ± 0.03, CI = [0.61, 0.72], low other-status: 0.58 ± 0.03, CI = [0.51, 0.64], *p* < 0.001, $\eta_{p}^{2}$ = 0.16).

Importantly, there was also a marginally significant three-way interaction between self-status, other-status, and offer level, *F*(1,92) = 3.22, *p* = 0.076, $\eta_{p}^{2}$ = 0.03. Further tests showed that for low offers, the two-way interaction between self-status and other-status was significant, *F*(1,92) = 5.913, *p* = 0.020, $\eta_{p}^{2}$ = 0.06, such that participants in low status evidenced a more robust difference in acceptance rates of low UG offers from high status proposers (0.23 ± 0.04, CI = [0.14, 0.32]) and low status proposers (0.45 ± 0.04, CI = [0.36, 0.53]), $\eta_{p}^{2}$ = 0.39, than when participants were in high status: low other-status: 0.30 ± 0.04, CI = [0.21, 0.39]; high other-status: 0.42 ± 0.04, CI = [0.34, 0.51], $\eta_{p}^{2}$ = 0.16. For high offers, there was no significant interaction between self- and other-status, *F*(1,92) < 1, *p* = 0.512. **Figure 2** presents acceptance rates for different offers.

### Discussion

Overall, Experiment 1 showed that self-status and other-status both influence responses to UG offers. Specifically, participants were more likely to accept low offers from high status proposers, and this effect was more robust for participants in the low self-status group than in high self-status group, which provides partial support for the Interactive Status Hypothesis. After attaining the social status ranking, high status participants experienced more positive emotions and less negative emotions than low status participants, validating the meaningfulness of the competition ranking. At the same time, low and high status participants evidenced no difference in emotions during UG, confirming past research showing that emotions cannot explain the effect of social status on UG decision-making (Hu et al., 2014, 2016). In the next experiment, to further confirm the Interactive Status Hypothesis and see whether the effect of social status on responses to UG offers exists when social status changes, we examined the effects of self-status and other-status on UG acceptance decisions using a within-subject design.

## Experiment 2

The purpose of Experiment 2 was to see whether the effects from Experiment 1 would remain across different contexts (i.e., when status changes). The general procedure was identical to Experiment 1, except that the same participants completed several rank-inducing sessions in which their social status was changed according to their performance in the sessions.

### Method

#### Participants

Once again, to determine the sample size, we used G^∗^Power 3 software (Faul et al., 2007), which showed that we needed a sample size of at least 24 for this study to have adequate power (1 – β > 0.80) to detect a medium-size effect (*f* = 0.30). Thirty undergraduate and graduate students from a local university in China participated in the study. Identical to Experiment 1, each participant was informed that the basic payment was around 30 Chinese yuan (about 5 USD) and additional monetary reward would fluctuate with their performance in the experiment (based on the random selection of 10 UG trial results). On average, participants received around 50 Chinese yuan (about 8 USD). Informed consent was obtained from each participant before the test. The experiment was conducted in accordance with the Declaration of Helsinki and was approved by the Ethics Committee of the School of Psychological and Cognitive Sciences, Peking University.

#### Design and Procedure

Experiment 2 had a 3 × 3 × 2 within-participant factorial design, with the first factor referring to the participant’s own social status (self-status: low vs. middle vs. high), the second factor referring to the proposer’s social status (other-status: low vs. middle vs. high), and the third factor referring to UG offer level (low vs. high). The star system and operational definition of low and high offer levels were the same as in Experiment 1.

In Experiment 2, the participant first competed in six rounds of the rank-inducing task (i.e., math competition). Then he or she was given a rank (high, middle, or low) according to his or her performance on the task. Following the ranking, the participant played UG with one proposer randomly drawn from the opponents. Different from Experiment 1, the participant was informed that after every several rounds of UG (36 rounds/ block), there would be a new block of the rank-inducing task. In other words, UG was interleaved between blocks of the rank-inducing task. Participants were also informed that the rank attained after each block of the rank-inducing task would pertain only to that particular block of the rank-inducing task and the ensuing block of UG. Each round of the rank-inducing task was composed of three easy and three difficult problems, which facilitated the manipulation of participant rank across rounds. The participants were also informed that the rank-inducing task had no direct relationship with UG. Partners in the rank-inducing task and UG were the same throughout the experiment.

In total, there were six blocks of the math competition, with six time-constrained math questions per block (36 in total, 10 s/question). The order of the ranks attained were counterbalanced across participants.

The second task was UG, which was identical to Experiment 1 (see Experiment 1 Method). There were six blocks of UG. We manipulated participant status (i.e., self-status: high vs. middle vs. low), proposer status (i.e., other-status: high vs. middle vs. low), and offer level (high vs. low), resulting in 18 critical conditions. Each condition included 12 trials.

Before the formal test, participants performed six trials of the math competition and 10 trials of UG to get familiar with the two tasks. To check the manipulation of social status, after the experiment, the participant was asked to indicate on a seven-point Likert Scale to what extent he/she perceived his/her status as higher (superior)/lower (inferior; 1 = much lower, 7 = much higher) than other players in the game when he/she was in each status condition. The participants were then debriefed, paid, and thanked for their participation.

### Results

#### Manipulation Checks

Among the thirty participants, one participant did not believe the experimental setup and was removed from further analysis, leaving 29 participants (20 females; mean age 20.7 years, *SD* = 1.4) for the following analysis.

The same manipulation check in Experiment 1 was used here. This post-experiment check indicated that the number of stars which was used to denote the participants’ rank in the math task strongly influenced their perception of social status. A one-way (star ranking: three vs. two vs. one) repeated-measures ANOVA on perceived status showed a significant main effect of star ranking, *F*(2,56) = 96.06, *p* < 0.001. Pairwise comparison on responses to the seven-point Likert Scale revealed that the participants perceived themselves to be in higher status when they obtained three stars in the math competition (mean ± SE, 5.28 ± 0.16, CI = [4.94, 5.61]) than when they obtained two stars (4.00 ± 0.09, CI = [3.82, 4.18]) and one star (2.41 ± 0.16, CI [2.08, 2.74]), *p*s < 0.001. Also, the participants perceived themselves to be higher in status when they obtained two stars than when they obtained one star, *p* < 0.001.

#### Behavioral Results

**FIGURE 3:**
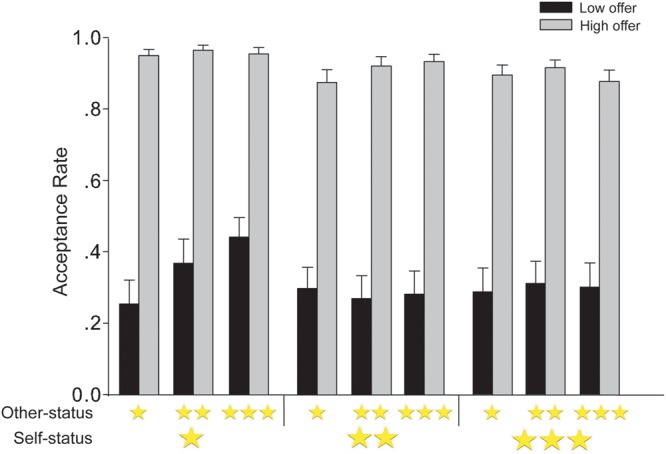
**The acceptance rate in Experiment 2 depicted as a function of self-status, other-status, and UG offer level.** One star = low status; two stars = middle status; three stars = high status. Error bars represent standard errors of the means.

We performed a 3 (self-status: high vs. middle vs. low) × 3 (other-status: high vs. middle vs. low) × 2 (offer level: high vs. low) repeated measures ANOVA on participants’ acceptance rates for different offers in UG (**Figure 3**). This analysis revealed a significant main effect of offer level, *F*(2,56) = 129.60, *p* < 0.001, $\eta_{p}^{2}$ = 0.82, with a lower acceptance rate for low offers (0.31 ± 0.06, CI = [0.19, 0.44]) than for high offers (0.92 ± 0.02, CI = [0.50, 0.71], *p* < 0.001). The main effect of self-status was also significant, *F*(2,56) = 14.21, *p* < 0.001, $\eta_{p}^{2}$ = 0.34, indicating that the acceptance rate was higher when the participants were in low status (0.66 ± 0.03, CI = [0.59, 0.72]) than when they were in middle (0.59 ± 0.04, CI = [0.51, 0.67], *p* < 0.001) or high status (0.60 ± 0.04, CI = [0.51, 0.68], *p* = 0.001). There was no difference between the acceptance rates when participants were in middle and high status (*p* = 0.740). The main effect of the proposer social status was also significant, *F*(2,56) = 11.45, *p* < 0.001, $\eta_{p}^{2}$ = 0.29, indicating that the acceptance rate was higher when the proposers were in high status (0.63 ± 0.04, CI = [0.55, 0.70]) than when they were in low status (0.59 ± 0.04, CI = [0.51, 0.67], *p* = 0.001). The acceptance rate was also higher for proposers in middle status (0.62 ± 0.04, CI = [0.55, 0.70] than for proposers in low status (0.59 ± 0.04, CI = [0.51, 0.67], *p* < 0.001). There was no difference between the acceptance rates when the proposers were in middle and high status, *p* = 0.605.

Similar to Experiment 1, Experiment 2 showed a significant interaction between other-status and offer level, *F*(2,56) = 5.09, *p* = 0.009, $\eta_{p}^{2}$ = 0.15. Further tests revealed that the acceptance rate for the low offers was lower when the proposers were in low status (0.28 ± 0.06, CI = [0.15, 0.41]) than when they were in middle status (0.32 ± 0.06, CI = [0.19, 0.45], *p* = 0.014) and high status (0.34 ± 0.06, CI = [0.22, 0.47], *p* = 0.001), and there was no difference between the acceptance rates for the low offers when proposers were in middle and high status, *p* = 0.332. For high offers, the acceptance rate of offers from low-status proposers (0.91 ± 0.02, CI = [0.86, 0.96]) was marginally lower than from middle-status proposers (0.93 ± 0.02, CI = [0.90, 0.97], *p* = 0.051), but was not different from high-status proposers (0.92 ± 0.02, CI = [0.88, 0.96], *p* = 0.783), and there was no difference in the acceptance rates of high offers from middle- and high-status proposers, *p* = 0.629.

We were most interested in the interaction between self-status, other-status, and offer level. The analysis revealed a three-way interaction, *F*(4,112) = 9.66, *p* < 0.001, $\eta_{p}^{2}$ = 0.26. To further analyze this three-way interaction, three separate two-way repeated-measures ANOVAs were conducted on participant acceptance rates when in low, middle, and high self-status. When participants were endowed with a low self-status, the main effects of offer level [*F*(1,28) = 115.01, *p* < 0.001, $\eta_{p}^{2}$ = 0.80] and other-status [*F*(2,56) = 15.31, *p* < 0.001, $\eta_{p}^{2}$ = 0.35] were significant, in addition to the interaction between offer level and other-status, *F*(2,56) = 15.36, *p* < 0.001, $\eta_{p}^{2}$ = 0.35. Simple effects tests showed that low status participants were less likely to accept low offers when they were offered by a low-status proposer (0.25 ± 0.07, CI = [0.12, 0.39]) than a middle- (0.37 ± 0.07, CI = [0.23, 0.51]) or high-status proposer (0.44 ± 0.05, CI = [0.33, 0.55]), *p*s < 0.001; participants in low status were slightly less likely to accept low offers from middle-status proposers (0.37 ± 0.07, CI = [0.23, 0.51]) than from high-status proposers (0.44 ± 0.05, CI = [0.33, 0.55]), *p* = 0.090. There was no difference between acceptance rates of high offers (*p*s = 1.00). When participants were endowed with a middle self-status, there was a main effect of offer level [*F*(1,28) = 134.73, *p* < 0.001, $\eta_{p}^{2}$ = 0.83], yet there was no main effect of other-status, *p* = 0.525, and the interaction between other-status and offer level was significant but had a smaller effect size than that of the low self-status condition *F*(2,56) = 3.50, *p* = 0.037, $\eta_{p}^{2}$ = 0.11. In addition, tests for simple effects showed no difference in acceptance rates for low or high offers given by low-, middle-, or high-status proposers, *p*s > 0.236. When endowed with a high self-status, the two main effects of offer level [*F*(1,28) = 115.06, *p* < 0.001, $\eta_{p}^{2}$ = 0.80] and other-status [*F*(2,56) = 3.56, *p* = 0.035, $\eta_{p}^{2}$ = 0.11] were significant, but the interaction was not, *p* = 0.275. Taken as a whole, the three-way interaction suggests that the effects of status and response decisions in UG were greatest when the participant was in a low-status position.

### Discussion

Overall, findings from Experiment 2 replicate the findings from Experiment 1 in a changing social hierarchy. These findings confirm that both self-status and other-status influence the responses to resource distribution. In addition, Experiment 2 provides strong support for the Interactive Status Hypothesis by showing that, in comparison with high and middle self-status, participants in low social status were more affected by the social status of others when deciding whether to accept or reject UG offers. In particular, when participants occupied low status, acceptance rates of low UG offers increased as a function of proposer social status, an effect not present when the same participants occupied middle or high status, which provides direct support for the Interactive Status Hypothesis, and which suggests that occupying a low status may elicit strategic, other-oriented behavior.

## General Discussion

The present study showed that the lower the status of the recipient, the more likely he/she was to accept low offers; additionally, the higher the status of the proposer giving a low offer, the more likely that offer was to be accepted. These two main effects suggest that both self- and other-status affect responses to resource distribution and confirm past studies suggesting that social status affects the acceptance of monetary allocations (Ball et al., 2001; Albrecht et al., 2013; Hu et al., 2014, 2016). Experiment 1 showed that participants were more likely to accept low offers from high status proposers, and this effect was more robust for participants in the low self-status group than in high self-status group. Experiment 2 largely replicated these findings in a changing hierarchy by showing that only in low status were participants more likely to accept low offers given by high status others. In addition, while in low status, participants’ acceptance rates of low offers increased as a function of other-status (**Figure 3**). These findings provide strong support for the Interactive Status Hypothesis.

Here, we propose two potential mechanisms underlying the interaction between self- and other-status on acceptance of low offers during resource distribution: one cognitive and one emotional. We found that participants were more affected by other-status while in low status than in high status, which supports past research on the unique cognitive and emotional effects of being endowed with low social status (De Kwaadsteniet and van Dijk, 2010; Kraus et al., 2011). On the one hand, from the *social cognitive perspective of social class*, while individuals from a low social class typically exhibit a contextual and externally oriented cognitive pattern, individuals from a high social class exhibit a solipsistic and individualistic cognitive pattern (Kraus et al., 2012). In light of this line of reasoning, low-status individuals should increase attention to others’ identities, thoughts, and actions (i.e., proposer social-status), and adjust their decisions accordingly (i.e., whether to reject low UG offers); high-status individuals should focus more on their own goals and interests (i.e., the inequality level of the offer) than others’ identity (i.e., proposers’ social-status).

On the other hand, from an emotional perspective, past research using a similar paradigm has found that participants viewing their own low rank status exhibit an increased P2 amplitude in electrophysiology, in comparison with when they view their own high status rank (Hu et al., 2014), which is thought to represent increased attention to unpleasant stimuli, especially those with a negative emotional valence (i.e., negativity bias; Carretié et al., 2001, 2004; Delplanque et al., 2004; Olofsson and Polich, 2007). Using this line of reasoning, one could infer that increased negative emotions may lead to an increased likelihood of accepting low offers during resource distribution. This would be in contrast with existing findings on the effects of negative emotions during UG, which have shown that priming negative emotions leads to an increase in rejection rates of UG offers (Harlé and Sanfey, 2007). However, these differences may be due to differences in the experimental design, as Harlé and Sanfey (2007) primed feelings of sadness using short movie clips, whereas our past (Hu et al., 2014) and current studies elicited interpersonal emotions. Given certain constraints of the current and past studies (i.e., UG emotions were measured offline), future research aimed at better understanding the potential explanatory role of these two accounts in explaining acceptance behavior would greatly benefit our understanding of the effect of social status on responses to resource distribution.

Social status is a relative construct that elicits changes in mindset from one context to the next. A professor may enjoy high status with his/her doctoral students and experience low status when meeting with the dean. Findings from Experiment 2, in which social status changes occurred within minutes of each other, suggest that individuals can enter new social status mindsets very quickly. Not only are adaptations to social status mindsets rapid, but these adaptations have meaningful influences on decision-making behavior with real economic consequences. One interesting question for future research is whether people experience social status differently depending on the status of their partners. For example, a low status participant could experience his/her low status differently when playing UG with a low status proposer than a high status proposer. Also, given the rapid adaptation to status-related mindset changes evidenced in Experiment 2 when participants were in a more passive role (i.e., responding to the offer of the proposer), one other interesting question for future research would be whether previous findings regarding the effects of social status are adaptive across contexts when the individual is in an active role, such as choosing between ethical and unethical behavior (e.g., Piff et al., 2012).

There are three additional points worth mentioning. First, a classic study by Knoch et al. (2006) shows that, under certain conditions, recipients in UG are able to consciously perceive an offer as unfair and still accept it. An interesting question would be whether or not participants in low status accepted low offers despite judging them as unfair. In the current study, post-experiment questions probing participants’ fairness judgments of varying UG offers showed no clear influence of social status on judgments of fairness, which suggests that the effects of perceived fairness may need to be tested online or implicitly (e.g., via skin conductance response). In Experiment 1, high and low status participants reported no difference in emotions during UG, which could suggest that feelings of fairness may have been affected by social status. As these findings would have interesting societal ramifications, future studies should analyze online feelings of both emotions and fairness to see what is underlying the increased likelihood of accepting low offers while in low status. These findings may also have interesting implications for the debate over whether disadvantaged individuals are more likely to accept unfair realities. For instance, System Justification Theory proposes that low status people are more likely to support the system as it is (i.e., status quo), despite inequality (i.e., status-legitimacy effect; Jost et al., 2004); however, recent work questions the robustness of the status-legitimacy effect (Brandt, 2013). If the feelings of social status from our study are similar to feelings of low social class, our findings provide indirect support for the status-legitimacy effect, as the behavior of participants in low status (i.e., acceptance rates of low UG offers increased as a function of proposer social status) would act to maintain/perpetuate the order of social status within the hierarchy.

Another interesting question for future studies is whether the findings from Experiment 2 would be present during hierarchy instability within one’s group. One important component in social hierarchies is their stability. In unstable hierarchies, interactions with other group members are more salient socially and behaviorally than in stable hierarchies (Zink et al., 2008), as high status members are striving to maintain their status, whereas low status members want to increase their status, leading to potential struggles for social status. Moreover, in stable social hierarchies, the greatest amount of stress is experienced by low status individuals, whereas in unstable hierarchies, high status members experience the greatest amount of stress in order to retain their position and settle conflict (Sapolsky, 2004, 2005). Given the importance of hierarchy stability and that Experiment 2 confirmed the Interactive Status Hypothesis in individuals whose social status changed across contexts, future studies would benefit from analyzing the robustness of these effects in stable and unstable hierarchies.

Finally, given that social status and power are similar yet distinct constructs (Magee and Galinsky, 2008), future studies should also consider whether the social status effects found in the current study have any influence on or could be explained by a perceived sense of power. In the current study, the endowment of social status led to no direct influence or control over the amount of money another individual received, hence the effects we obtained are best interpreted as social status and not as power.

## Conclusion

The current study showed that social status is a critical factor in responses to resource distribution. During economic interactions, low status individuals are more sensitive to the status of others. In particular, when occupying low status, acceptance of low UG offers increases as a function of others’ social status, whereas high status individuals’ behavior is far less affected by others’ social status. The findings from the current study could have important implications for understanding the behavior and mindset of individuals in a social hierarchy, showing that low status individuals’ acceptance of low offers may be strategic and related to the status of the other individual in question, and that high status individuals’ behavior may be less attuned to contextual information during decision making in social interactions. Moreover, in general, support for the Interactive Status account also has interesting implications not only for social psychology (i.e., relative social status influences behavior), but also for behavioral economics (i.e., acceptance of low offers are context-dependent), and evolutionary psychology (i.e., reasons behind acceptance of low offers in a social hierarchy). Our results may also help us to understand responses to resource distribution in status-related interactions in the workplace.

## Author Contributions

PB, JH, EvD, and XZ designed the experiment; PB, JH, XW collected the data; PB, JH, XW, EvD, and XZ wrote the manuscript.

## Conflict of Interest Statement

The authors declare that the research was conducted in the absence of any commercial or financial relationships that could be construed as a potential conflict of interest.
